# Yeast Hydrolysate Inhibits Lipid Accumulation via Regulation of Lipid Accumulation-Related Genes in a *Drosophila* Model of High-Sugar Diet-Induced Obesity

**DOI:** 10.3390/ijms242216302

**Published:** 2023-11-14

**Authors:** Nari Kim, Yejin Ahn, Kayoung Ko, Boyun Kim, Kisoo Han, Hyung Joo Suh, Jewon Jung, Ki-Bae Hong

**Affiliations:** 1 Department of Integrated Biomedical and Life Science, Graduate School, Korea University, Seoul 02841, Republic of Korea; 84hurizia@korea.ac.kr (N.K.); ks.han@cremar.co.kr (K.H.); suh1960@korea.ac.kr (H.J.S.); 2 Research Group of Functional Food Materials, Korea Food Research Institute, Wanju 55365, Republic of Korea; a.yejin@kfri.re.kr; 3 Department of Food Science and Nutrition, Jeju National University, Jeju 63243, Republic of Korea; lv007@jejunu.ac.kr; 4 Department of SmartBio, Kyungsung University, Busan 48434, Republic of Korea; boyunism@gmail.com

**Keywords:** yeast hydrolysate, body composition, high sugar, *Drosophila*, behavior, MTCA

## Abstract

The increasing frequency of processed food consumption has led to the higher ingestion of sugar, increasing the risk of chronic diseases, such as obesity. Yeast hydrolysates (YHs) inhibit body fat accumulation. However, the action mechanism of YH in relation to high-sugar diet-induced obesity is still unclear. Therefore, this study aimed to evaluate the biological effects of YH on lipid accumulation and verify behavioral changes and carbohydrate metabolic gene regulation in high-sugar diet-fed fruit flies. Adult male flies (*Drosophila melanogaster*; 2–5 days old) were exposed to 20% sucrose for obesity induction. In high-sugar-fed *Drosophila*, the effect of YH was compared with that of yeast extract. The effects of YH on body conditions and lipid droplet size were quantified and analyzed. Behavioral factors were evaluated by analyzing circadian rhythm patterns and neurotransmitter content, and a molecular approach was used to analyze the expression of metabolism-related genes. Dietary supplementation with YH did not reduce total sugar content, but significantly decreased the triglyceride (TG) levels in *Drosophila*. A behavioral analysis showed that the total number of night-time activities increased significantly with YH treatment in a dose-dependent manner. In addition, YH effectively regulated the gene expression of insulin-like peptides related to carbohydrate metabolism as well as genes related to lipogenesis. The TG content was significantly reduced at a YH concentration of 0.5%, confirming that the active compound in YH effectively suppresses fat accumulation. These findings support that YH is a potential anti-obesity food material via regulating carbohydrate metabolism in *Drosophila*.

## 1. Introduction

Carbohydrates, including sugars, are the most important source of energy for the human body and are essential nutrients for all physical and intellectual activities while imparting sweetness and texture to food. However, the continued consumption of refined sugars, mainly sucrose and glucose–fructose syrups, is associated with the rapid absorption of glucose in the blood, and sugar overconsumption can lead to a variety of health problems that involve metabolic and psychiatric disturbances [1,2]. In addition, the overconsumption of energy-dense foods leads to a lower quality of life, a reduced immune system, and several debilitating conditions, including heart disease, diabetes, and obesity [3,4,5,6]. Therefore, low-calorie content is becoming popular among consumers, and the food ingredient industry is focusing on the development and sale of anti-obesity materials. Moreover, owing to growing health concerns, consumers have started using alternative anti-obesity materials such as semisynthetic, synthetic, and natural substances.

*Saccharomyces cerevisiae*, baker’s and brewer’s yeasts, are known to have free radical scavenging, stress-resistant, and immune activation capacities and are essential microorganisms in food biotechnology. Further, the beneficial effects of selenium and carboxymethyl-glucan-rich hydrolysates from yeast on psoriasis-like dermatitis and baroreflex sensitivity have been reported in in vitro and in vivo experiments [7,8]. In clinical trials using chemical hydrolysates of yeast, β-glucan–chitin–chitosan showed significant changes in body weight, waist circumference, and abdominal fat accumulation without adverse effects [9]. Although various physiological and molecular approaches have been developed to elucidate the mechanisms involved in weight control using yeast hydrolysates (YH), the exact mechanisms and supporting evidence remain inconclusive.

Human metabolic genes are conserved in the fruit fly, *Drosophila melanogaster*, allowing the functional analysis of orthologues implicated in human diet and diet-related diseases. Secretion of insulin and signaling of fat body (FB) and insulin-producing cells (IPCs) in *Drosophila* show similarities in the balance between the stored and circulating forms of energy. FB acts as a key sensor that responds to the nutritional status of *Drosophila* and relates nutritional availability to systematic growth and metabolism [10]. Owing to its similarity in biochemistry and genetic background to humans, *Drosophila* is a feasible model for analyzing changes in glucose uptake, fat accumulation, and energy metabolism with dietary intake.

*D. melanogaster*, an experimental model, has a brain nervous system, internal digestion organs, adipocytes, and insulin secretory cells and shares approximately 70% of human disease-related genes. It is actively used in metabolic physiology and disease fields. Diet-induced obesity in *Drosophila* is related to reactive oxygen species, oxidative stress regulators, cell regulation (Forkhead Box O, FOXO), and lifespan. In recent years, there has been a growing interest in safe and low-risk food materials owing to the adverse effects caused by chemical synthetic substances. In particular, low-molecular-weight yeasts and hydrolysates developed using enzyme and ultrafiltration technologies are known to inhibit body fat accumulation, stress, and anxiety. However, the detailed action mechanism of YH in relation to high-sugar diet-induced obesity has not yet been studied. Therefore, this study aimed to identify the targets of YH that could induce inhibition of de novo synthesis/re-esterification such as fat oxidation, inhibition of accumulation, improvement of lipid concentration, and alteration of obesity-related genes using a *Drosophila* model of high-sugar diet-induced obesity. 

## 2. Results

### 2.1. Effects of YH on Body Composition

**Figure 1:**
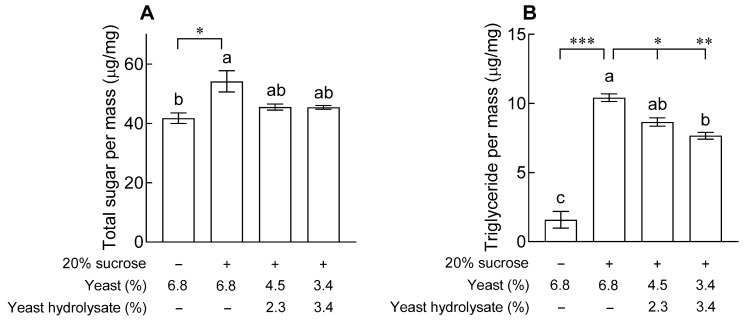
Effects of yeast hydrolysate on levels of (**A**) total sugar and (**B**) triglyceride in fruit flies. The given values are the mean ± standard error of the mean from 90 fruit flies per group. The letters indicate significant differences at *p* < 0.05 by using Tukey’s test, and the symbols indicate significant differences at * *p* < 0.05, ** *p* < 0.01, and *** *p* < 0.001 by using Dunnett’s test.

As shown in Figure 1, the total sugar content in the bodies of the control group treated with 20% sucrose was significantly higher than that in the normal group (Figure 1A, *p* < 0.05). Additionally, the total sugar content of fruit flies on a diet containing YH showed no significant difference compared to the normal group and control group treated with 20% sucrose. The difference in TG content significantly increased with 20% sucrose (Figure 1B, *p* < 0.001), and the group exposed to YH decreased in a dose-dependent manner compared to the control group, with a more significant decrease at a concentration of 3.4% (*p* < 0.01).

### 2.2. Effects of YH on Area Size of Single Lipid Droplet

**Figure 2:**
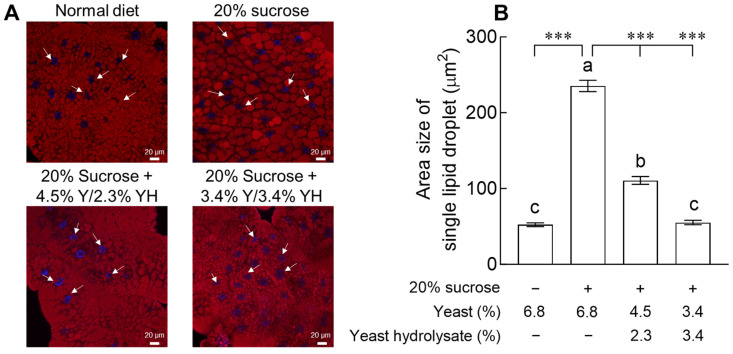
Effects of yeast hydrolysate (YH) on area size of single lipid droplet in the abdominal fat body of fruit flies. (**A**) Lipid droplets (red) and nuclei (blue) are shown for flies and (**B**) areas of lipid droplets in fat body cells were quantified by using microscopic analysis. The values are the mean ± standard error of the mean from 60 fruit flies per group. The letters indicate significant differences at *p* < 0.05 by using Tukey’s test, and the symbols indicate significant differences at *** *p* < 0.001 by using Dunnett’s test.

The effects of YH on the area size of single lipid droplets in larvae exposed to 20% sucrose for 1 week are shown in Figure 2. Due to 20% sucrose exposure, the area size of single lipid droplets in the control group significantly increased 4.5-fold than that in the normal group (Figure 2B, *p* < 0.001). In the group in which the yeast content was replaced with 2.3% YH, the area size of a single lipid droplet significantly increased compared to that in the normal group, but a significant decrease was observed at 47.08% compared to that in the control group (*p* < 0.001). In larvae exposed to 3.4% YH, the area size of the single lipid droplet showed a significant decrease compared to that of the group treated with 20% sucrose (*p* < 0.001). The area size decreased to a level similar to that of the normal group. The results demonstrated that YH affected the area size of a single lipid droplet in a dose-dependent manner.

### 2.3. Effects of YH on Locomotor Activity and Gamma-Aminobutyric Acid Content

**Figure 3:**
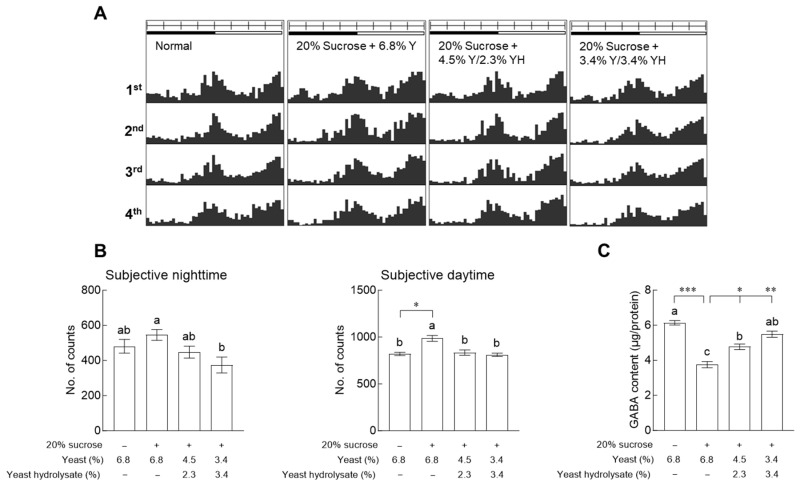
Effects of yeast hydrolysate (YH) on locomotor activity in fruit flies. (**A**) Typical actograms of individual normal flies and flies exposed to YH, by dose. Average activity in a 30-minute interval was calculated over 4 days. Black/white bars on top of the actograms indicate dark (22:00 to 10:00) and light (10:00 to 22:00) phases. (**B**) Activity during subjective night-time and daytime. (**C**) Gamma-aminobutyric acid (GABA) concentration in fruit fly heads. The values are the mean ± standard error of the mean from 60 fruit flies per group. The letters indicate significant differences at *p* < 0.05 by using Tukey’s test, and the symbols indicate significant differences at * *p* < 0.05, ** *p* < 0.01, and *** *p* < 0.001 by using Dunnett’s test.

Figure 3 shows the changes in circadian rhythm patterns and neurotransmitter levels. In the case of subjective night-time, the locomotor activity of the group exposed to 3.4% YH was significantly reduced compared to that of the control group exposed to 20% sucrose (Figure 3B, *p* < 0.05). In addition, during the subjective daytime, locomotor activity was significantly increased in the control group than in the normal group (Figure 3B, *p* < 0.05), and the treatment groups exposed to YH did not show any significant difference from the normal group. In the subjective daytime, the content of gamma-aminobutyric acid (GABA), an inhibitory neurotransmitter that affects behavior and sleep, was significantly reduced in the control group, which ingested only 20% sucrose, compared to the normal group (Figure 3C, *p* < 0.001). When exposed to YH, the GABA content of adult fruit flies increased in a dose-dependent manner and was significantly different from that of the control group (*p* < 0.05 and *p* < 0.01).

### 2.4. Effects of YH on Drosophila Insulin-like Peptides and Unpaired 2 mRNA Expression

**Figure 4:**
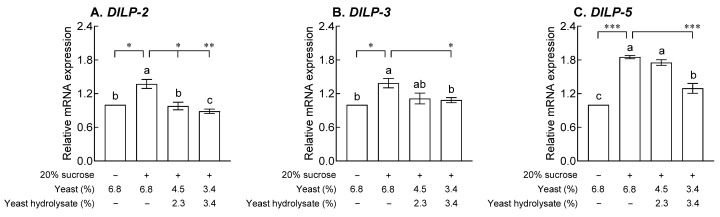
Effects of yeast hydrolysate on (**A**) *Drosophila* insulin-like peptide (*DILP*)*-1*, (**B**) *DILP-3*, and (**C**) *DILP-5* mRNA expression in fruit flies. The values are the mean ± standard error of the mean from 100 fruit flies per group. The letters indicate significant differences at *p* < 0.05 by using Tukey’s test, and the symbols indicate significant differences at * *p* < 0.05, ** *p* < 0.01, and *** *p* < 0.001 by using Dunnett’s test.

**Figure 5:**
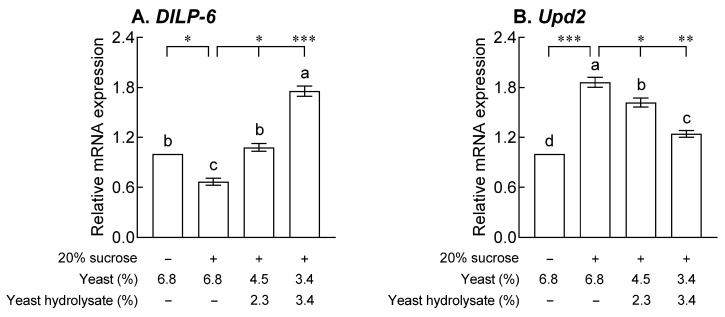
Effects of yeast hydrolysate on (**A**) *Drosophila* insulin-like peptide *(DILP)-6* and (**B**) unpaired 2 (*Upd2*) mRNA expression in fruit flies. The values are the mean ± standard error of the mean from 100 fruit flies per group. The letters indicate significant differences at *p* < 0.05 by using Tukey’s test, and the symbols indicate significant differences at * *p* < 0.05, ** *p* < 0.01, and *** *p* < 0.001 by using Dunnett’s test.

Figure 4 shows the effect of YH on the levels of genes *Drosophila* insulin-like peptide *(DILP*)*-2*, *-3*, and *-5* secreted by IPCs, which bind to insulin receptors in peripheral tissues to promote growth and nutrient utilization and regulate blood sugar and lipid metabolism. Compared to the normal group, the control group showed significantly increased mRNA expression of *DILP-2*, *-3*, and *-5* due to 20% sucrose exposure (*p* < 0.05 and *p* < 0.001). *DILP-2* mRNA expression decreased in a dose-dependent manner compared to that in the control group after YH exposure (Figure 4A, *p* < 0.05 and *p* < 0.01). In addition, 3.4% YH exposure resulted in a significant decrease in *DILP-3* and *-5* mRNA expression compared to that in the control group (Figure 4B,C, *p* < 0.05 and *p* < 0.001), and *DILP-3* expression was reduced to a level similar to that in the normal group. The mRNA expression of *DILP-6* and unpaired 2 (*Upd2*) released from FB adipocytes to communicate energy availability in the brain is shown in Figure 5. In the control group, the expression of *DILP-6*, which reduces the secretion of *DILP-2*, *-3*, and *-5* in IPCs and modulates lipid metabolism, was significantly reduced compared to that in the normal group (Figure 5A, *p* < 0.05). Exposure to YH increased *DILP-6* mRNA expression in a concentration-dependent manner compared to that in the control group (Figure 5A, *p* < 0.05 and *p* < 0.001), and exposure to 3.4% YH resulted in a significant difference in *DILP-6* expression than that in the normal group. In addition, the expression of *Upd2*, which increases the secretion of *DIP-2*, *-3*, and *-5* and promotes systemic growth and fat storage upon 20% sucrose exposure, was significantly increased (Figure 5B, *p* < 0.001), and *Upd2* expression decreased in a YH dose-dependent manner compared to that in the control group (*p* < 0.05 and *p* < 0.01).

### 2.5. Effects of YH on Lipid Metabolism-Related mRNA Expression

**Figure 6:**
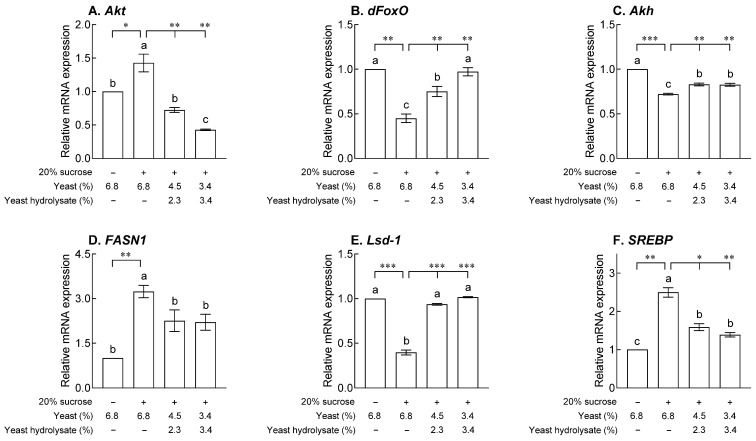
Effects of yeast hydrolysate on (**A**) Akt kinase (*Akt*), (**B**) forkhead box, sub-group O (*dFoxO*), (**C**) adipokinetic hormone (Akh), (**D**) fatty acid synthase 1 (FASN1), (**E**) lipid storage droplet-1 (Lsd-1), and (**F**) sterol regulatory element binding protein (*SREBP*) mRNA expression in fruit flies. The values are the mean ± standard error of the mean from 100 fruit flies per group. The letters indicate significant differences at *p* < 0.05 by using Tukey’s test, and the symbols indicate significant differences at * *p* < 0.05, ** *p* < 0.01, and *** *p* < 0.001 by using Dunnett’s test.

Figure 5 shows the effects of YH on the mRNA expression patterns of lipid metabolism, metabolic homeostasis, and lipogenesis in adult flies exposed to 20% sucrose. The expression of Akt kinase (*Akt*), a gene that inhibits expression in the nucleus by being involved in the phosphorylation of forkhead box, sub-group O (*dFoxO*), in the control group exposed to 20% sucrose was significantly increased compared to that in the normal group (Figure 6A, *p* < 0.05). YH decreased the concentration-dependent expression of *Akt* mRNA compared to that in the control group (Figure 6A, *p* < 0.01). Expression of *dFoxO*, a gene that inhibits lipogenesis, was significantly reduced in the control group than that in the normal group after 20% sucrose exposure (Figure 6B, *p* < 0.01). In the YH group, *dFoxO* mRNA expression was significantly higher than that in the control group (*p* < 0.01). The expression of the adipokinetic hormone (*Akh*) gene, associated with the homeostasis of storage lipids, was significantly reduced by 20% sucrose than that in the normal group (Figure 6C, *p* < 0.001) and significantly increased in the groups exposed to YH (*p* < 0.01). The control group exposed to 20% sucrose showed significantly increased fatty acid synthase 1 (*FASN1*) mRNA, involved in glycogen metabolism and triglyceride biosynthesis, expression (Figure 6D, *p* < 0.01) and significantly decreased lipid storage droplet-1 (*Lsd-1*) mRNA, involved in facilitating lipolysis, expression compared to the normal group (Figure 6E, *p* < 0.001). In the groups exposed to YH, *Lsd-1* mRNA expression was significantly higher than that in the control group (Figure 6E, *p* < 0.001). In the case of the sterol regulatory element binding protein (*SREBP*) gene, which is a lipogenesis factor, the control group showed a significant increase compared to the normal group (Figure 6F, *p* < 0.01) and a significant decrease in the groups exposed to YH (Figure 6F, *p* < 0.05 and *p* < 0.001).

### 2.6. Effects of Active Compound from YH on Lipid Accumulation

**Figure 7:**
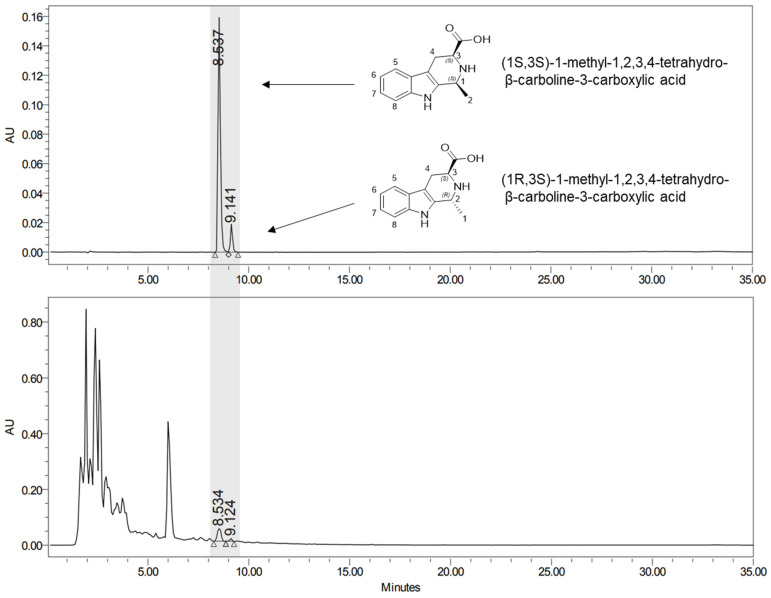
Chromatograms of 1-methyl-1,2,3,4-tetrahydro-β-carboline-3-carboxylic acid standard and yeast hydrolysate sample using high-performance liquid chromatography. Agilent ZORBAX SB-C18 (5 μm, 4.6 × 150 mm) was used. UV absorption was measured at 277 nm and 25 °C.

**Figure 8:**
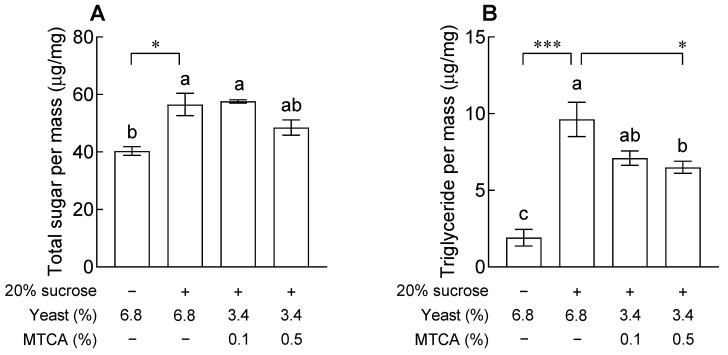
Effects of 1-methyl-1,2,3,4-tetrahydro-β-carboline-3-carboxylic acid (MTCA) on levels of (**A**) total sugar and (**B**) triglyceride in fruit flies. The values are the mean ± standard error of the mean from 90 fruit flies per group. The letters indicate significant differences at *p* < 0.05 by using Tukey’s test, and the symbols indicate significant differences at * *p* < 0.05 and *** *p* < 0.001 by using Dunnett’s test.

The content of 1-Methyl-1,2,3,4-tetrahydro-β-carboline-3-carboxylic acid (MTCA), which is estimated to be the active compound of YH, was 0.55 mg/g in YH (Figure 7). The fat accumulation inhibitory effect of MTCA, measured by adding 0.1 and 0.5% MTCA along with yeast extract to a 20% sucrose diet, is shown in Figure 8. As it was difficult to analyze the administration of the active compound of YH within the *Drosophila* model, 0.1% and 0.5% MTCA were administered. The total sugar and TG contents in the 20% sucrose group were significantly higher than those in the normal group, and the 0.1% MTCA group showed no significant difference from the control group, which had a high sucrose content. When 0.5% MTCA was provided, the TG content was significantly reduced compared to that in the group provided with 20% sucrose. Thus, MTCA contained in YH was verified as an active compound that can inhibit fat accumulation.

## 3. Discussion

Although carbohydrates, including sucrose, are essential for life and activity maintenance, excessive sugar intake is a major cause of various diseases in adults. Therefore, the interest in and demand for alternative sugars and food materials that can inhibit fat accumulation are increasing. Among these, YH is a suitable functional food material that can suppress blood sugar levels and regulate lipid metabolism. In this study, fruit flies, *D. melanogaster*, were used as a model to analyze major homeostatic mechanisms and investigate potential changes in response to hyperglycemia.

In the present study, in order to verify the effect of YH in inhibiting lipid accumulation, we attempted to match the protein content ratio of the standard medium by replacing the reduced yeast with YH. We investigated the effects of YH and found that flies showed a dose-dependent difference in the body fat storage and area size of a single lipid droplet (Figure 1 and Figure 2). According to a previous study, the content of TG in the hemolymph and whole body of flies on a 30% sucrose diet increased 2.5- and 2.0-fold, respectively, compared to those in flies on a 2% sucrose diet [11]. Thus, *Drosophila* may also use the conversion of glucose-derived carbons into fat body-stored TG as a protective measure when exposed to a high-calorie carbohydrate-rich diet [12]. In addition, restriction of dietary yeast, the main source of proteins in fruit fly diets, affects the expression of fatty acid synthesis and oxidation genes, thereby increasing fat accumulation [13]. In general, foods with hypoglycemic and antilipidemic effects may improve insulin sensitivity by minimizing the changes in blood glucose levels and reducing insulin secretion. Moreover, previous clinical trials showed significant changes in weight, BMI, and body fat mass in women aged 20 to 60 years old and obese adults when YH was administered for 8 to 10 weeks, and these results were reported to be related to changes in energy intake [14,15].

Based on the measurement of circadian rhythm patterns and GABA content (Figure 3), the total activity and neurotransmitter content in the subjective daytime period of the 20% sucrose group (control group) were significantly different compared to those of the normal and YH groups. An increase in glucose levels after the intake of nutrients is detected by the fat body; consequently, signals reach the brain and IPC [16]. In addition, systemic DILP and insulin-like growth factor signaling are involved in metabolism and longevity, as well as in regulating behaviors and cognitive functions [17]. This change in behavior is caused by *Up2*, the functional homolog of human leptin, acting on cytokine receptor Domeless, which blocks GABA release by activating JAK/STAT signaling in GABAergic neurons [16]. A previous study showed that metabotropic GABA_B_ receptors are expressed in IPCs and that GABA is involved in the inhibitory regulation of DILP levels and metabolism [18].

Similar to mammalian insulin, DILP secretion from IPCs is controlled by nutrient availability. DILPs perform a variety of physiological functions, including the regulation of organism growth, life span, lipid storage, and carbohydrate metabolism. As shown in Figure 4 and Figure 5, exposure to YH significantly altered the mRNA expression of *DILPs* and *Upd2* compared to that in the control group. Depending on nutrient availability, *DILPs* are involved in the regulation of nutrient utilization, and recent studies have defined the roles of *DILP*-2, 3, 5, and *6* in longevity regulation via carbohydrate and lipid metabolism [19]. *DILP-6* is expressed extensively in fat body cells during the larval and adult stages and is related to adipose tissue and brain endocrine functions that regulate metabolic and longevity phenotypes. *DILP-6* transcription is regulated by *dFOXO* and is associated with the decreased expression of other *DILPs*, such as *DILP-2*, *3*, and *5* [20]. Taken together, these results suggest that increased *DILP-6* mRNA expression may have a regulatory effect on *DILP-2*, *-3*, and *-5* mRNA expression in the YH-exposed groups. In addition to direct carbohydrate sensing, IPCs are also involved in energy metabolism and nutrient regulation via multiple signals derived from the fat body. The fat body conveys information about carbohydrates through at least three mechanisms such as secretion of *Upd2*, TGF-β/activin ligand Dawdle, and CCHamide-2 [16,21].

The effects of YH on the target genes associated with lipid and glucose metabolism in adult *Drosophila* were analyzed using qRT-PCR (Figure 6). The metabolisms of glucose and fatty acids, the major sources of energy, are intertwined and regulated, and excessive carbohydrates are converted into fatty acids and cholesterol through de novo lipid biosynthesis pathways. In addition, abundant circulating carbohydrates are related to the anabolic actions of insulin, resulting in glucose uptake, glycogen synthesis, and lipolysis in the fat bodies of *Drosophila* [22]. Numerous studies have focused on the multitarget molecular mechanisms of pharmaceutical agents and non-pharmacological remedies involved in reducing lipid synthesis, inducing lipid decomposition, regulating lipid absorption, and controlling appetite. Compared to vertebrates, *Drosophila* has a full set of lipogenic enzymes as well as functionally similar underlying metabolic and signaling pathways involved in carbohydrate and lipid metabolisms, which are highly related to numerous metabolic diseases [23,24]. In high-sugar fruit flies, similar gene networks, which are conserved from flies to mice and humans, regulate obesity susceptibility [25]. Suppression of insulin-like signaling and fatty acid metabolism are the main mechanisms related to dietary calories and longevity extension in fruit flies [26]. Regarding the regulation of the insulin signaling pathway and lipogenesis, the reduced expression of *Akt*, *dFoxO*, *Akh*, *FASN1*, *Lsd-1*, and *SREBP* in *Drosophila* (on a high-sugar diet) exposed to YH alleviated fat accumulation.

The results of the isolation of MTCA, an active compound of YH, and its inhibitory effect on fat accumulation in the *Drosophila* model are presented in Figure 7 and Figure 8. MTCA, which exists in two diastereomers (1S,3S and 1R,3S), is present in commercial fruits and garlic (*Allium sativum* L.) and has radical-scavenging activities [27,28]. MTCA, an active compound isolated from garlic, reduced the expression of adipogenic genes and inhibited adipogenesis in 3T3-L1 cells [29]. In addition, Kim et al. reported that YH and MTCA suppress adipogenic lipid storage by downregulating SREBP- and NADPH-synthesizing genes in 3T3-L1 cells [30].

## 4. Materials and Methods

### 4.1. Materials

YH was prepared as previously described, with slight modifications [30]. *S*. *cerevisiae* cultured by Choheung Chemical (Seoul, Republic of Korea) was treated with proteolytic enzymes, and YH of 10 kDa or less were recovered using a 10 kDa molecular weight cutoff membrane (Nanofiltration System, SEC Co., Ltd., Gunpo, Republic of Korea) and spray dried (EINSYSTEM Co., Ltd., Seoul, Republic of Korea). The contents of the total crude protein of YH and the active substance, MTCA (Sigma-Aldrich, St. Louis, MO, USA), were 550 and 0.55 ± 0.04 mg/g, respectively.

### 4.2. D. melanogaster Stock and Maintaining Condition

Fruit fly experiments were performed using the wild-type Canton-S strain distributed by the Bloomington *Drosophila* Stock Center at Indiana University (Bloomington, IN, USA). Flies were maintained in an incubator at 24 ± 1 °C, 60% relative humidity, 12:12 h light: dark cycle and reared on standard medium (4.3% cornmeal, 5.0% sucrose, 6.8% yeast, 1.0% agar, 0.45% propionic acid, and 0.2% p-hydroxybenzoic acid methyl ester). Single treatments of high-sugar (20%) and YH (2.3% and 3.4%) were dissolved in distilled water and mixed in a standard medium for the analysis of body composition, neurotransmitter content, and target mRNA expression. In addition, the yeast content (safe-instant yeast, Lesaffre, France) added to the standard medium was changed according to the YH content.

### 4.3. Analysis of Body Composition

To analyze the body composition of the fruit flies, live adult male flies were euthanized under CO_2_ anesthesia. They were homogenized in 300 μL phosphate-buffered saline (PBS) with 0.05% Triton-X using an automated tissue processor. The homogenized samples were centrifuged at 12,000× *g*, 15 min, and 4 °C, and the supernatant was stored at −80 °C. To compare the total body composition between the normal and sample-treated groups, the total glucose, protein, and triglyceride (TG) contents were measured using fly homogenates. Total sugar and reduced sugar contents were determined using the method described by Miller [31] with some modifications, using glucose as a standard. Hemolymph glucose levels were measured using a glucose kit (Sigma-Aldrich). The protein content was measured using a BCA Protein Assay kit (Thermo Fisher Scientific, Waltham, MA, USA). The TG content was measured by using a Triglyceride Quantification kit (Sigma-Aldrich), and the result was expressed as μg/mg of body mass.

### 4.4. Lipid Droplet Staining

The changes in the area size of single lipid droplets were quantified as previously described [32]. For lipid droplet staining, the larvae were dissected in PBS and fixed in 4% paraformaldehyde at room temperature for 30 min. The tissues were rinsed with PBS and incubated in PBS containing 0.05% Nile Red (Sigma-Aldrich), BODIPY (Invitrogen, Carlsbad, CA, USA), and Oil Red O. DAPI was used to stain the nuclei. The stained samples were mounted on 75% glycerol, and the lipid droplet size was quantified by microscopic analysis. The areas of the ten largest lipid droplets per cell (50 fat body cells in total) were measured.

### 4.5. Behavioral and Neurotransmitter Assay

Changes in circadian rhythm-related behavior were analyzed using a previously reported method [33]. The *Drosophila* Activity Monitoring System (TriKinetics, Waltham, MA, USA, version 10.1.10) was used to analyze the differences in behavior when the yeast hydrolysate was substituted for yeast in a medium containing high sugar. Individual fruit flies were acclimated to the tube and incubator for 24 h at 24 ± 1 °C, and all behavioral changes were recorded every minute using an infrared detector in constant darkness for 4 days. Data were analyzed using Actogram J software (version 1.51), and the total number of activities recorded daily was calculated as the sum of each activity divided by the subjective night and daytime periods.

The GABA content in *Drosophila* heads was determined by using high-performance liquid chromatography (HPLC) fluorescence detection. All experiments were performed in triplicate, with 50 flies per replicate. Fly heads from each group were homogenized in 0.1 M TCA as previously described [34] and samples were centrifuged at 12,000× *g* for 10 min. The supernatant was filtered through a 0.45 μm polyvinylidienefluoride (PVDF) syringe filter, and GABA levels were determined by the Waters AccQ-Tag system utilizing a Waters 2475 Multi λ Fluorescence detector. Samples were injected into an HPLC system (Waters e2695 Separations Module, Milford, MA, USA) consisting of a Waters AccQ-Tag column (3.9 mm × 150 mm) and column heater (37 °C).

### 4.6. Gene Expression Analysis

To extract total RNA, whole bodies of flies were homogenized and isolated using 1.0 mL TRIzol^®^ reagent (Invitrogen) according to the manufacturer’s protocol. After genomic DNA was removed using RQ1 RNase-free DNase I (Promega, Madison, WI, USA), cDNA was synthesized using oligo-d(T) primers and SuperScript III Reverse Transcriptase (Invitrogen). Quantitative real-time polymerase chain reaction (qRT-PCR) was performed on the resulting cDNA using a Power Taqman PCR Master Mix kit (Applied Biosystems, Foster City, CA, USA). The gene expression levels were calculated after normalization to the expression of the reference gene ribosomal protein L32 (RpL32: NM_001144655.3) using the ΔΔCt method [35]. Information for target genes used in qRT-PCR was: *DILP*-2 (NM_079288.3), *DILP*-3 (NM_140103.3), *DILP*-5 (NM_206315.2), *DILP*-6 (NM_001201609.1), *Upd2* (NM_001370039.1), *Akt* (NM_001300424.1), *dFoxO* (NM_001275628.1), *Akh* (NM_079194.2), *FASN1* (NM_001144306.2), *Lsd-1* (NM_001275954.1), and *SREBP* (NM_001275135.1).

### 4.7. Analysis of Active Compound Contents

The active compound analysis was used to quantify MTCA contents. Yeast hydrolysate solution was filtered through a 0.45 μm PVDF membrane, and an HPLC system with a PDA detector was used for the analysis (Waters). A C-18 column (Agilent ZORBAX SB-C18, 5 μm, 4.6 × 150 mm, Agilent Technologies, Santa Clara, CA, USA) was used for the separation at 277 nm. Water and acetonitrile, both containing 0.1% formic acid, were adjusted to a flow rate of 0.5 mL/min. A gradient of 5% to 65% acetonitrile in water for 30 min was used for the separation. MTCA contents were calculated using a standard MTCA (Sigma-Aldrich) curve.

### 4.8. Statistical Analysis

Statistical analyses were conducted using the Statistical Package for Social Sciences Statistics 18 (IBM Corporation, Armonk, NY, USA). The experimental data were represented as mean ± standard error of the mean. Significant differences for each sample group were evaluated using a one-way analysis of variance with Tukey’s multiple-range test. Dunnett’s multiple comparison test was used to determine the significance of intergroup differences against a control group.

## 5. Conclusions

This study verified the functionality of the food material using a high-sugar diet-fed *Drosophila* and analyzed gene expression related to carbohydrate and lipid metabolisms through the administration of YH, which is known to be involved in weight loss and appetite control. When yeast, the main ingredient in the standard medium, was replaced with YH, the TG content and area size of the single lipid droplet in *Drosophila* (obesity induced by 20% sucrose) were significantly reduced. In addition, the regulation of DILP signaling by exposure to YH caused changes in neurotransmitter content and subjective daytime locomotor activity. In addition, TG accumulation was inhibited upon exposure to MTCA, an active compound in YH. Taken together, YH was confirmed to be a substance that inhibits fat accumulation by influencing the expression of genes related to carbohydrate and lipid metabolism as well as neurotransmitter content and behavioral changes.

## Data Availability

The data presented in this study are available on request from the corresponding author.
